# Development and Acceptability of a Co-Produced Online Intervention to Prevent Alcohol Misuse in Adolescents: A Think Aloud Study

**DOI:** 10.2196/humanfactors.4452

**Published:** 2015-07-29

**Authors:** Emma Louise Davies, Jilly Martin, David R Foxcroft

**Affiliations:** ^1^ Department of Psychology, Social Work and Public Health Oxford Brookes University Oxford United Kingdom; ^2^ Department of Psychology University of Sheffield Sheffield United Kingdom

**Keywords:** adolescents, alcohol, intervention development, prevention, think aloud

## Abstract

**Background:**

The prototype willingness model (PWM) may offer an appropriate basis for explaining and preventing adolescent alcohol misuse. An intervention was developed using a co-production approach, and consisted of an online quiz featuring 10 questions linked to the PWM.

**Objective:**

This study sought to determine the acceptability and relevance of the intervention content to young people, to incorporate their feedback into a final version.

**Methods:**

A qualitative think aloud study with follow-up semistructured interviews was undertaken with 16 young people aged 11-15 (50%). Transcripts were analyzed using thematic analysis.

**Results:**

The following 3 main themes relating the acceptability of the intervention were identified: “challenging expectations of alcohol education”; “motivations for drinking or not drinking,” and “the inevitability of drinking.” Participants found the intervention appealing because it was counter to their expectations. The content appeared to reflect their experiences of social pressure and drinking encounters. There was evidence that a focus on drinker/nondrinker prototypes was too narrow and that because adolescents perceived drinking as inevitable, it would be challenging to enact any plans to resist pressure to drink.

**Conclusions:**

An online intervention based on the PWM has the potential to engage and interest adolescents. A wide range of alcohol prototypes should be targeted and a focus on short-term harms should ensure that the intervention is credible to young people.

## Introduction

### Overview

Underage alcohol consumption is higher in the United Kingdom than in other parts of Europe [1] and evidence suggests teenagers aged 11-15 who consume alcohol are at risk of short-term harm [2,3] and later dependence [4]. National surveys suggest that the number of young people in England aged 11-15 who report ever having tried alcohol is falling [5]; however, other evidence suggests that those who do drink tend to consume harmful quantities [6,7]. This evidence points to a need for the development of effective intervention measures to reduce adolescent alcohol misuse and associated harms.

Many interventions aimed at adolescents rely on popular models, such as the theory of planned behavior (TPB) [8], which rest on assumptions of reasoned decision making and intention-driven behavior. However, there is often a discrepancy between what people intend to do and what they actually do [9,10]. This “intention-behavior gap” is particularly problematic in explaining adolescent health risk behaviors [11]. In support of this, a recent meta-analysis suggested that adult alcohol intentions might be better accounted for by the TPB than adolescent alcohol intentions [12]. This may be because adolescence is characterized by high levels of impulsivity, which is linked to risk-taking behaviors, such as drinking alcohol [13], and tends to peak between the ages of 13 and 19 [14,15]. Drinking at this age tends to occur in social situations where peer influences are strong [16,17] and may provide a challenge to the developing brain [18].

Some evidence suggests that theory-based health behavior change interventions tend to have larger effect sizes than those that are not theory based [19]. However, a recent meta-analysis suggests that some theory-based interventions may fail to appropriately target each construct within the selected theory, and furthermore, not all behavior change techniques (BCTs) are linked to theory [20]. It is therefore essential to identify an appropriate theoretical basis for an intervention to reduce alcohol misuse in adolescents, and to ensure that it is appropriately applied within the intervention.

### Prototype Willingness Model

**Figure 1:**
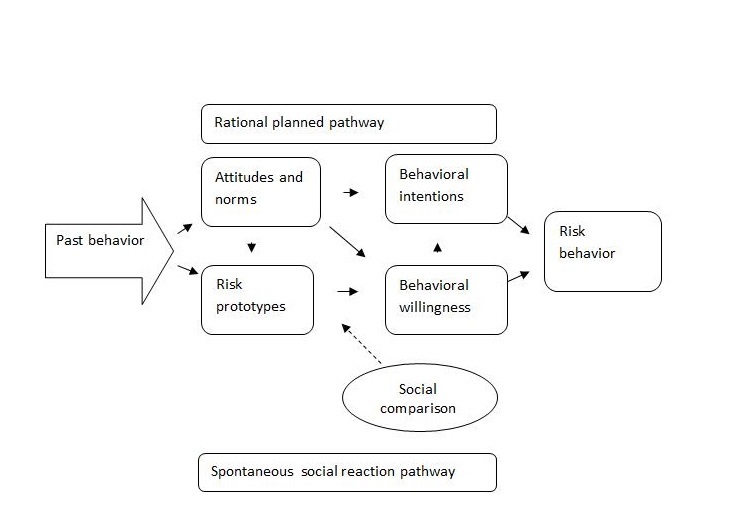
The prototype willingness model. Adapted from Gerrard et al [28].

The prototype willingness model (PWM) [21,22] accounts for adolescent health risk taking on the basis that this type of behavior is driven by social reactions to risk-conducive situations, as well as intentions (Figure 1). In common with other dual process models, there are 2 routes to behavior within the PWM: the first, a rational, planned route via intentions, and a second reactive pathway, which is a faster, more spontaneous route, operating outside of conscious control [22]. The spontaneous pathway considers that for young people, risky behaviors tend to occur in a social context and are often unplanned [23]. Within this pathway, the images or “prototypes” that young people have about typical people of their age who drink or abstain from drinking are influential for “willingness” to consume alcohol. This is due to the importance of self-image and social comparison in adolescence [17].

Previous research has shown that the PWM is able to offer a good explanation for risk behaviors, such as alcohol consumption, in young people [24-26]. Studies have also shown that the PWM may offer a suitable basis for an intervention (eg, substance misuse [27] and physical activity [28]). A number of studies have applied this model to alcohol consumption in the United Kingdom, by university students [29] and adolescents aged 16 [30]. However, there is less research that specifically examines the PWM in relation to preventing alcohol misuse in young adolescents, under the age of 16, in the United Kingdom. This study therefore sought to develop an intervention based on the PWM to explore its application to this population.

### Intervention Development

Intervention development has been conceptualized within a number of phases by the Medical Research Council [31]. The “development” phase within this framework covers the important process of identifying the evidence base and ensuring the intervention is clearly linked to theory, a step that is sometimes neglected [32]. To specify a clear pathway through the development phase, we set out a number of steps at the outset of the project, starting with a scoping focus group study and a survey [33,34]. A co-production approach was taken, involving input from adolescents, teachers, and parents as key stakeholders in the intervention at different stages of its development. Co-production aims to acknowledge and empower young people (and other stakeholders) through collaboration in the intervention development process [35].

There were 2 important findings from the focus group study. First, it showed that that young people in the United Kingdom were able to describe drinker and nondrinker prototypes that potentially could be targeted in an intervention. Second, the findings also suggested a distinction between “planned” drinking by older participants (aged 16-17) and “unplanned” drinking in younger participants (aged 11-13) [34]. A survey of 178 adolescents aged 11-17 was then built on these findings by exploring the relationship between prototypes, willingness, intentions, and alcohol consumption. The survey results suggested that young people aged 11-15 were likely to be a more appropriate age group for an intervention targeting prototypes and willingness than those aged 16 or 17. Furthermore, an exploratory factor analysis suggested that targeting prototype characteristics that were related to “sociability” might be an appropriate focus within the intervention [33].

Although there has been a drive in recent years to classify BCTs according to theoretical and behavioral features, at the time of development, no clear BCTs related to the PWM had been specifically defined and agreed. Thus, within the development of this project, we identified techniques used in 8 existing PWM interventions, comparing them with a taxonomy of BCTs [36] and identifying if they adequately reflected the assumed change processes in the PWM. This process was evaluated in a Delphi study, reported elsewhere [37], which resulted in 4 BCTs being identified that were relevant to the social reaction pathway of the PWM. Table 1 presents the identified BCTs and how they relate to the PWM.

**Table 1:** Logic model to specify behavior change techniques, processes, and outcomes for prototype willingness model intervention in the social reaction pathway.

Input (behavior change technique)	Process in the model	Outcome
Present information on other people’s drinking to reduce perception of drinker prototype as the norm to enhance similarity to nondrinker.	Images are often based on misperceptions. Similarity to prototype drinker is strongly related to willingness and drinking.	Drinker prototype similarity decreases. Corrects norm misperception.
Present a positive nondrinker and or negative drinker prototype and enhance similarity to nondrinker.	Target prototype favorability and similarity. Enhance positive features of nondrinker. Present negative image of drinker.	Drinkers and drinking are less favorable and less similar to self. Nondrinkers and nondrinking more favorable and more similar to self.
Teach awareness of social/environmental cues to behavior (that reactive or unplanned is more risky).	Spontaneous influences on behavior may occur when young people do not plan to drink.	Young people are aware of reactive nature of their behavior.
Provide examples of how other young people resist social pressure in social situations.	Reduce unplanned behavior and decrease willingness to drink.	Young people are able to recognize and deal with social pressure themselves.

It is important to ensure that the content and format of an intervention are matched to the preferences of the intended recipients [38]. Discussions from the focus group study suggested that the participants might not be receptive to a classroom intervention delivered by a teacher [34]. Adolescents who attended schools that took part in the focus groups and surveys were consulted in the process of selecting the most appropriate means of delivering the intervention within the classroom. They reported that they preferred to engage with interactive online materials rather than written information. Furthermore, other evidence highlighted the benefits of using computer games to enhance learning within a school context [39] and that online interventions might be a useful means of reaching younger populations [40]. Research with young people suggests a familiarity with using the Internet for schoolwork, and that 46% of young people complete quizzes online [41]. A quiz format was selected as an appropriate mode of delivering the intervention because it required engagement with the content and has been used in other interventions targeting adolescents [42]. At this point, we named the intervention “The Alcohol Smart Quiz” (ASQ) in consultation with adolescents.

The quiz consisted of 10 multiple-choice questions linked to the identified BCTs. In line with previous PWM intervention research [43,44], the information in the quiz was presented as originating from a survey of adolescents who were of the same age as the intended recipients. The answers were provided as explanations from other young people talking about their own experiences. The first 5 questions targeted alcohol prototypes. For example, there were questions that require the participant to select characteristics of the typical drinker or nondrinker who is of the same age as they are. The second 5 questions targeted social pressure and unplanned drinking. This part included questions and answers where young people describe that they resist pressure to drink by making a plan in advance of what they will say if they are in a social situation where alcohol is present. The quiz materials are available from the main author on request.

### Think Aloud

In a think aloud study, participants are required to talk out loud about what they think as they complete a task or a questionnaire. Think aloud interviews have been widely used in psychology as a method of cognitive interviewing [45,46]. For example, French et al [45] used this method to explore what participants understood when reading TPB questionnaires. Think aloud interviews have more recently been used by intervention designers who saw the potential of this method in contributing to an understanding of how users interpret theoretical techniques and relate intervention content to their own experiences [47,48]. This method is also useful for ensuring that the terminology used is understandable to particular samples [47]. It therefore offers an appropriate method of gaining feedback from young people.

The overall aim of this study was to explore adolescent views about the ASQ intervention to determine the acceptability and relevance of the content to young people, and to incorporate their feedback into a final version, as part of the development process.

## Methods

### Participants

There were 16 participants; 8 boys and 8 girls aged from 11-15 (in year groups 6-11 in the English school system). The participants attended 12 different schools in the South East of England. Interviews were conducted and analyzed until data saturation was reached. Participants were recruited through advertisements to parents and offered a £10 voucher to thank them for taking part. The study received ethical approval from Oxford Brookes University (reference number 120619).

### Materials

A paper version of the intervention was constructed using a printed and laminated PowerPoint slide to represent each page of the website. This was presented on a document stand so that participants could flip between pages. A paper version was used so that changes could be made to the content following the study before utilizing funds to build the website. Paper versions of online interventions have been used in similar studies [47]. The pages represented the quiz questions, and answers are presented with pictures of young people of a similar age depicted as giving answers to the questions (see Multimedia Appendix 1). Participants were informed that once the intervention is available online, videos of real people would be used to provide the answers.

### Think Aloud Interviews

Interviews took place in a quiet room on university premises and consent was obtained from both the parent and the participant. At the start of the session, the researcher checked the parent had talked about the study to the participant and if they were happy to proceed. The interviewer read out some standardized instructions and demonstrated thinking aloud by completing a similar task, which involved answering questions in a quiz about favorite foods. Participants then worked though each page of the intervention and were prompted to tell the interviewer what they thought of each question. This was followed with some semistructured interview questions to explore factors related to intervention acceptability (Textbox 1). Interviews lasted between 25 and 40 minutes, were audio recorded, and then fully transcribed.

**Semistructured interview schedule of follow-up questions used in think aloud study.**

Overall views about the quiz

- What did you think of the quiz?
- Was it easy to understand what you have to do?
- What would you think if you were given this quiz to play at school? At home?
- What improvements could you make?

What did you think about the answers?

- Some of the questions talked about how drinkers and nondrinkers were described—what did you think about the answers?
- What do you think about the answers on peer pressure?
- There were some questions about making plans—what did you think about them?

Learning about alcohol

- What do you think that other people of your age would think about this?
- Is a quiz or a game a good way to find out information about alcohol?
- Have you seen anything similar? Can you tell us about it?
- Are there any other good ways to find out information about alcohol?

Ending questions

- Do you have anything else you would like to add about the materials you have seen, or the topic we have been talking about?

### Analysis

Transcripts were subjected to thematic analysis using the stages set out by Braun and Clarke [49]. During familiarization, the transcripts were read and re-read and ideas for codes were noted. An initial set of 36 codes was identified and applied across the dataset. These codes were reviewed during the search for themes resulting in some being merged or renamed. Other codes were combined to form overarching themes relating to the dataset. An initial thematic map consisting of 3 main themes (relating to “expectations about alcohol education,” “perceptions of drinking and drinkers,” and “experiences with alcohol”) was generated. Each theme had a number of related subthemes. This thematic map was developed through testing with the data and discussion between all authors until an agreement was reached on a final set of themes relating to “challenging expectations of alcohol education,” “motivations for drinking or not drinking,” and “the inevitability of drinking” (Table 2).

## Results

### Themes and Subthemes

In line with other intervention development research employing the think aloud method [48], this paper focuses on the themes in relation to positive and negative features of the ASQ, because of their implications for intervention development. Supporting quotes for each theme and subtheme are presented using pseudonyms and indicating the sex and age of the participant.

**Table 2:** Themes and subthemes related to aspects of the acceptability of the Alcohol Smart Quiz identified in analysis of think aloud interviews.

Main theme	Subtheme
Challenging expectations of alcohol education	A different mode of delivery
	This is not “the usual message”
Motivations for drinking or not drinking	Experiences of pressure
	Consequences of drinking
	Perceptions of drinkers
The inevitability of drinking	Normative nature of “drinking as cool”
	Barriers to making plans in the real world

### Challenging Expectations of Alcohol Education

#### Overview

The theme “challenging expectations of alcohol education” encapsulates the participants’ responses to the ASQ as something unexpected when compared with their experiences of alcohol education in school, as well as what they had been told by parents and other adults. These expectations appeared to be related to both the format and the content of the intervention.

#### A Different Mode of Delivery

The online mode of delivery and the quiz format appeared to be well received by the participants in this study. In particular, they liked that it was presented as an online game with interactive features.

I like it because, if it is just something written down, then that would be boring, but having it as a game is more interesting.

Archie, m, 14

It was also favorably compared with school-based alcohol education, where a teacher might stand up at the front of the class and present information.

If you get a teacher to talk to the students about alcohol, then no-one is going to say anything because they are with their friends.

Lucas, m, 15

There was also support for using video clips of young people presenting the answers to the quiz once the ASQ had been put on a website because participants felt that people of the same age would be easier to relate to than a teacher. Furthermore, presenting the information as a quiz with a number of possible options appeared to be a positive feature.

If you just tell someone a fact, they won’t think for themselves, but here if you get it wrong then it makes you think.

Matthew, m, 13

#### This Is Not “the Usual Message”

Intervention content seemed to be different to the information that the participants had expected. They appeared surprised to find out that the number of young people aged 11-15 who reported drinking alcohol has fallen in recent years. This unexpected content may have challenged their preconceptions that “everybody drinks.” As this was the first question, it seemed to set the scene that they were not going to hear the usual messages about drinking and that this might be something different.

Quite often, in school, you will get told “don’t drink, or you will die” sort of thing, which isn’t that helpful.

Kasia, f, 14

The idea of making plans in advance to deal with a situation also seemed to be unexpected and something that participants found interesting.

Things about peer pressure, they just tell you not to give in, but this is something that you could actually do.

Vicky, f, 13

There was also information that seemed surprising in some of the questions about making plans to avoid drinking. In particular, most participants were apparently unaware about the amount of calories in a bottle of wine when this was mentioned in a quiz question about planning to refuse alcohol:

I didn’t even know you could get calories in a drink!

Muna, f, 11

Overall, it appeared that the topics covered in the quiz questions had the potential to capture the participants’ attention, in particular because they were in contrast to their expectations.

If something surprises you about a subject, then it probably makes you think twice.

Matthew, m, 13

### Motivations for Drinking or Not Drinking

#### Overview

The theme “motivations for drinking or not drinking” draws together the complex reasons behind alcohol consumption for the young people in this study. As expected, based on the literature, peer pressure was a common feature of the participants’ talk. The consequences of drinking appeared to be described in a negative way, but this did not seem to discourage the participants or their friends. Nondrinkers tended to be described in a negative way.

#### Experiences of Pressure

A positive feature of the ASQ was that the content of the quiz questions and the scenarios described appeared to relate to the participants’ experiences with alcohol and social pressure. Most of the participants reported feeling some pressure in relation to alcohol, as well as smoking. The presence of other people was often acknowledged as a reason for drinking.

If there’s a lot of people around you and they’re all doing it and then they’re saying to do it then you are more likely to do it than if you were on your own and there was beer in the fridge.

Lucas, m, 15

If everyone else was doing it then you wouldn’t want to be the odd one out.

Alice, f, 12

There was also evidence of further distinction evident in the participants’ experiences of pressure, which could be either explicit and involve direct coercion

Oh that’s so stupid and babyish if you don’t.

Emily, f, 12

They say “don’t be a pussy” and stuff.

Natalia, f, 14

Or could be implied pressure

When other people start drinking and smoking even if they don’t actually pressure you, you will be pressurised even though they are not saying anything to you...because you know at some point you will lose out of the group by not doing the same thing.

Muna, f, 11

If you are at a party and everyone else is doing it, they could be quite persuasive, you would feel boring or antisocial.

Vicky, f, 13

This apparent distinction between explicit and implied pressure is important to take into account when describing social pressure to drink with the intervention.

#### Consequences of Drinking

A number of questions talked about the consequences of drinking, and this was another aspect that appeared to be reflective of participants’ experiences. These were mainly the short-term negative outcomes, such as being sick or suffering an injury. Some participants talked about friends who had been to hospital to have their “stomach pumped out” or who had come into contact with the police. Participants appeared to focus on the negative physical or social consequences of drinking alcohol; for example, some participants talked about attending parties and seeing people who had too much to drink:

A girl I know didn’t eat for three days before the party, she wanted to be skinny or something, yeah she was sick all night long.

Natalia, f, 14

I don’t think people know their limits, or when to stop.

Rachel, f, 15

Consequences relating to short-term embarrassment also seemed important.

Having an embarrassing photo, that’s a good answer, because everyone has Facebook now, it is likely that you would do that.

Chloe, f, 12

#### Perceptions of Drinkers

Quiz questions about prototypical nondrinkers described them as sociable, confident, and independent. Participants tended to agree with this answer and some talked about other positive characteristics of nondrinkers in response.

Like really cool and strong and you know being able to not drink if lots of people are drinking.

Emily, f, 12

I don’t necessarily think they’d use these three words [sociable, confident, independent] they’d use other ones like chilled, relaxed and things like that.

Lucas, m, 15

However, there was some evidence within the transcripts that suggested that nondrinkers would be viewed negatively by other people.

At parties you know everyone joins in but then there’s some people that just decide not to and then they just get sort of judged in a way sometimes cos they are the odd one out.

Alice, f, 12

The findings also suggest caution in the way that drinker prototypes are presented. Drinkers appeared to be perceived as cool by many of the participants:

There’s a system, if someone is not cool, you can’t hang out with them if you want to be cool too, and people think the drinkers are cool.

Jon, m, 11

However, there appeared to be caveats to this. Heavy drinking and drunkenness tended to be described using negative language.

People who have got really drunk at parties, that’s not cool, it looks a bit sad.

Kasia, f, 14

However, drinking a little was usually described as normal by the older participants.

I think it is normal to have a drink, maybe a glass of cider or something, alcohol in moderation is fine.

Matthew, m, 13

Other comments revealed that it might be important to tailor drinker and nondrinker descriptions carefully.

You can’t stereotype people as those who go out and those that stay at home, I am somewhere in between.

Sam, m, 15

These quotations suggest that a focus on moderate drinking compared with heavy or binge drinking might be more appropriate for the intervention.

### Inevitability of Drinking

#### Overview

Regardless of the positive response from these participants toward the ASQ, there was a sense of drinking as an inevitable feature of teenage life. The theme “the inevitability of drinking” reflects the findings that alcohol was appeared to be perceived as something “cool,” and as such, resisting its draw might be challenging.

#### Normative Nature of “Drinking as Cool”

The perception of drinking as cool was frequently identified in participants talk about the intervention as they completed the quiz questions, possibly because it was prohibited.

I think probably because it’s actually not allowed to people like older than about 18 so it’s kind of like, to be honest if someone’s banned something then it makes it all the more cool if you do it.

Jon, m, 11

In the shops they have a special section for all of this tobacco and stuff like that so I think that makes it, oh look, I’m special, I’m going here too.

Muna, f, 11

It possible that this “coolness” contributed to participants’ reasons for trying alcohol for the first time. Although they acknowledged the power of peer pressure, many participants suggested that their reasons for initially trying alcohol were out of curiosity for this “cool” and “forbidden” substance.

I wanted to see what it tasted like, I was just really curious cos I mean I’d tried like wine and things from a young age, it tastes horrible, it’s like rat poison and suddenly like you try it at about 14 and it’s rocket fuel, it’s brilliant, and so then you are like oh, damn I want to try all these things, it’s like an adventure of discovery.

Sam, m, 15

I would have thought that quite a lot of people would be peer pressured into it a bit but also that people would be kind of curious.

Vicky, f, 13

These comments suggest that it is important to take into account that young adolescents are likely to be curious about alcohol. It may be challenging to alter their perception of it as a “cool thing to do,” and so a clear focus on reducing harm appears to be more appropriate than a focus on avoiding alcohol.

#### Barriers to Making Plans in the Real World

Although the idea of making plans to deal with pressure to drink in social situations was unexpected and positively received, there were many pieces of evidence in the transcripts that indicated participants felt unsure about whether this could really be applied in real life. First, the issue of whether you would actually be able to enact a plan:

I think the idea of making a plan is quite a good idea but I think it’s a different matter whether you actually stick to the plan...it is quite unlikely that you will actually stick to it in the situation.

Kasia, f, 14

Then there was the issue that the situational pressure may prove too powerful

Um, if they think it is cool to drink they will laugh at you and won’t listen.

Joe, m, 12

Even if you made a plan in advance, you could still be tempted.

Alice, f, 12

Overall, it appears that participants believed formulating plans in advance to deal with social pressure was an interesting concept, but not something that they could realistically enact in a real-life situation. This might be because the social pressure in a given situation would overwhelm any intended plans. Participants came up with a number of alternatives to making plans to avoid alcohol that they thought would be useful for drinking less in alcohol-related scenarios.

Maybe if you had like a friend who was like responsible...if you had an older friend then sort of arrange with them saying if I am not there at that time then I’m drunk so come and find me, something like that.

Vicky, f, 13

This suggests that it may be possible to encourage young adolescents to focus on plans to avoid harms from drinking, rather than plans to avoid or refuse alcohol.

## Discussion

### Findings

This paper presented themes and subthemes from the analysis of think aloud interviews with 16 young people. The findings demonstrate that the ASQ had a number of features that demonstrated high levels of acceptability and relevance to the target population. An intervention delivered in schools that is different to what is expected has the potential to capture young people’s attention and engage them in the topic. Moreover, because the content of the ASQ related to participants’ experiences of drinking and pressure, this has the potential to enhance its credibility. In particular, the focus on short-term potential harms such as social embarrassment and increased calorie consumption reflects genuine concerns.

However, the identified themes also revealed important areas where improvements to the planned intervention should be considered. First, there is a need to consider how to describe alcohol prototypes in the ASQ. Participants disagreed about how they would describe the typical person of the same age as them who drank alcohol. Younger participants described them as “sad” or “stupid” and others who were older described them as “normal.” However, the evidence from the transcripts suggested that a “drunk” prototype would be seen as negative. The perception of nondrinkers was also mixed; negative views were that they were boring or the odd one out. However, some of the participants also said that nondrinkers were sensible or relaxed, which were more positive descriptions. There is little research that explores young adolescents’ perceptions of nondrinkers. Research with university students suggests that nondrinkers struggle to be accepted socially, and that a negative perception is normative in the United Kingdom [50]. In our previous focus group study with younger age groups, we found that nondrinkers were perceived as unusual or boring [34].

Second, there was evidence to suggest that although participants were generally positive about the idea of making plans to avoid pressure, they were concerned about whether this would actually be effective in practice. The planning questions in the ASQ were based on implementation intentions, or “if-then” plans [51]. However, it is possible that the plans were not presented in the most optimal manner with the ASQ. They were simply presented as examples and did not explicitly encourage the participants to develop and contemplate their own personal plans.

One way to improve the application of technique in this intervention could be to use volitional help sheets. In a previous study, Arden and Armitage [52] supplied a list of potential situations within which undergraduate students might be tempted to binge drink, together with possible solutions they could use to avoid this behavior. Linking the situations with the solutions created the personal if-then statements, which are central to implementation intentions [51]. Similarly, in another study, students were given options of things that they could say to refuse drinks [53]. The options included saying “no thanks, I do not want to get drunk” or “no thanks, I am watching my weight.” Participants were also asked to detail the time and place at which they would enact these plans. These studies were successful in reducing binge drinking in student participants [52,53].

It is possible, therefore, that young people will be able to make successful plans even if they think that it would not work, as long as they could be convinced to do so. Studies that have explored younger adolescents’ ability and motivation to make successful plans about alcohol consumption have not been identified. However, a recent study has demonstrated a successful application of implementation intentions to alcohol use with 16-year-old school pupils [54]. Thus, a major improvement to the ASQ would be to provide a range of potential scenarios and refusal options with the quiz questions and to explore the effectiveness of this approach.

Finally, it is important to consider how drinking behavior is perceived by the intended population. Drinking was perceived to be cool because it was forbidden, and therefore, it gave adolescents status among their peers. This supports Crossley’s [55] suggestion that risk-taking behaviors symbolize a transgression of social rules and rebellion for young people. Although some participants who had tried alcohol said that they had done so out of curiosity and not because they thought it would make them appear cool, it was clear that this was an important driver in maintaining the behavior. Trying alcohol for the first time was seen as inevitable during the teenage years. Evidence shows that 90% of 15-16-year olds in the United Kingdom have tried alcohol at least once and half have engaged in heavy episodic drinking (>5 drinks) in the last 30 days [1]. Within the ASQ, the quiz questions discuss short-term harms such as being sick, or having an embarrassing photo uploaded to a social media site, which appeared to be in line with participants’ concerns. However, further improvements could be made to ensure that the aspects of the ASQ that target prototypes are credible. Because of the inevitability of drinking for these participants, a focus on abstinence and enhancing nondrinker prototypes is probably an unrealistic goal. These findings suggest that in UK adolescents a “nondrinker” prototype target may not be seen as credible. A better focus could perhaps be to look at heavy or binge drinkers compared with moderate drinkers. Some research in the Netherlands identified different dimensions of drinker prototypes such as “tipsy,” “moderate,” and “heavy” drinkers [56], but this was in an older sample. Within British culture, drinking during the teenage years appears to be seen as part of growing up [34] and once adolescents reach young adulthood, many engage in heavy drinking [57]. Other qualitative research has highlighted the importance of tailoring intervention content to the intended population, suggesting a focus on encouraging young people who drink not to get “too drunk”[38].

Participants in this study described their perception of how peer pressure operates and revealed it to be a complex interplay between perceptions of drinking and the reactions you might receive if you did not drink. There also was a sense of inevitability about pressure to drink, which highlights the importance of this aspect of the intervention.

### Study Limitations

Limitations to this study should be taken into account. First, the participants were sampled through convenience, and were self-selected via their parents. Although the sample size is appropriate for this type of study, a wider range of young people may have been able to bring different issues to light in relation to the intervention. Furthermore, it would be useful to explore differences by age and sex in detail, which was not possible with this sample size. Parents were required to bring participants to the university and meet the interviewer leading to a possibility that the participants doubted the anonymity of what they said. In addition, it is important to note the influence of the researcher; participants may have been attempting to provide socially desirable answers. However, all efforts were made to ensure participants were assured of confidentiality, and they were not asked directly to discuss their own drinking behavior. Furthermore, participants’ responses to the ASQ were most likely influenced by their previous experiences of alcohol education in school. The think aloud section of the interview always took place first, and thus it is possible that the content of the ASQ influenced the participants’ responses to the follow-up questions. Furthermore, their reported attitudes and perceptions may well have been primed by the intervention content. Although we developed the ASQ to be delivered online, for the purposes of illustration, this study used a paper version. This alternative mode of delivery may not reflect the exact findings of our online version of the intervention, designed to enhance its appeal, which will feature videos and interactive content.

This study was conducted in the United Kingdom, where drinking rates among adolescents tend to be higher than in most other European counties and the United States [1]. While this limits the generalizability of the findings, it is important to develop culturally relevant intervention programs as well as to explore the application of popular theories, such as the PWM, across different cultures and contexts.

### Implications

The think aloud method meant that the content and format of the planned intervention could be tested with young people to explore their views before a trial. Increasingly, the value of conducting qualitative work before and alongside randomized controlled trials is being acknowledged [38,58,59] and the benefits of co-producing interventions are recognized. Although this method has been used to test other online interventions aimed at adults [47,48,59], no similar studies have been identified that have done so to test an alcohol misuse intervention with adolescents. This study has therefore demonstrated that this method can be used to obtain feedback from this population, and generate detailed discussions on the topic.

In conclusion, there are a number of specific implications of this study for improving the ASQ. The quiz format was well received but the final version should consider how it will be delivered in a classroom setting, to build on the positive features identified by the participants. The findings of this study suggest 3 main areas of focus for improvements.

First, the range of prototypes described in the quiz needs to be widened. Presenting a negative drunk prototype, rather than a negative drinker prototype, may be a more appropriate focus. Second, it is important to enable young people to enact plans to avoid harmful consequences of drinking. Finally, although the intervention does consider the complex perceptions of drinking as cool and how peer pressure affects young people’s decisions, it appears that pressure was an inevitable experience for these participants. Further work may be needed to explore the most effective means of delivering credible intervention messages both within the current intervention and more widely within an adolescent population.

## Appendix

Multimedia Appendix 1 Example screenshots of the Alcohol Smart Quiz intervention used within the think aloud study.

## Abbreviations

ASQ: Alcohol Smart Quiz
BCTs: behavior change techniques
PWM: prototype willingness model
TPB: theory of planned behavior
