# Supplementary material for: Development and Acceptability of a Co-Produced Online Intervention to Prevent Alcohol Misuse in Adolescents: A Think Aloud Study
Source: JMIR Hum Factors. 2015 Jul 29;2(2):e13. doi: 10.2196/humanfactors.4452 (PMC4797700; doi:10.2196/humanfactors.4452)
Supplement: Multimedia Appendix 1 [file humanfactors_v2i2e13_app1.pdf]

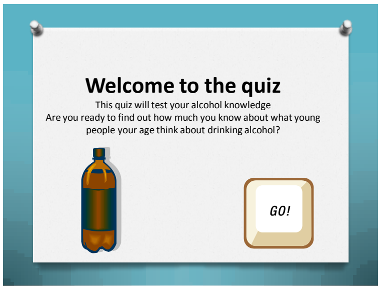
Welcome to the quiz
This quiz will test your alcohol knowledge
Are you ready to find out how much you know about what young people your age think about drinking alcohol?
GO!

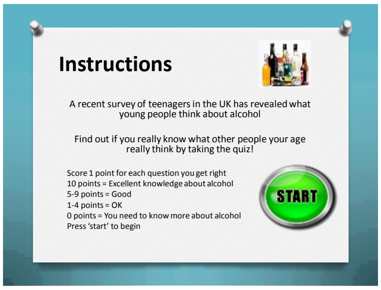
Instructions
A recent survey of teenagers in the UK has revealed what young people think about alcohol
Find out if you really know what other people your age really think by taking the quiz!
Score 1 point for each question you get right
10 points = Excellent knowledge about alcohol
5-9 points = Good
1-4 points = OK
0 points = You need to know more about alcohol
Press 'start' to begin
START

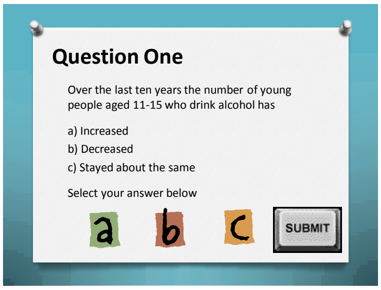
Question One
Over the last ten years the number of young people aged 11-15 who drink alcohol has
a) Increased
b) Decreased
c) Stayed about the same
Select your answer below
a
b
c
SUBMIT

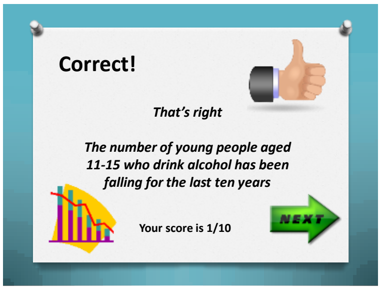
Correct!
That's right
The number of young people aged 11-15 who drink alcohol has been falling for the last ten years
Your score is 1/10
NEXT

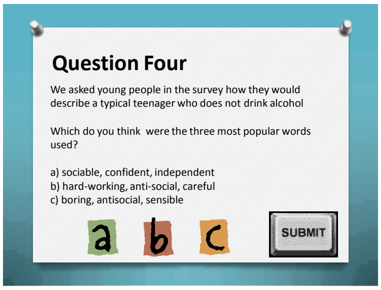
Question Four
We asked young people in the survey how they would describe a typical teenager who does not drink alcohol
Which do you think were the three most popular words used?
a) sociable, confident, independent
b) hard-working, anti-social, careful
c) boring, antisocial, sensible
a
b
c
SUBMIT

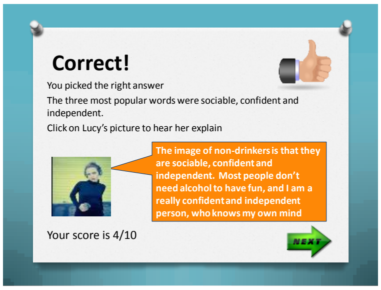
Correct!
You picked the right answer
The three most popular words were sociable, confident and independent.
Click on Lucy's picture to hear her explain
The image of non-drinkers is that they are sociable, confident and independent. Most people don't need alcohol to have fun, and I am a really confident and independent person, who knows my own mind
Your score is 4/10
NEXT

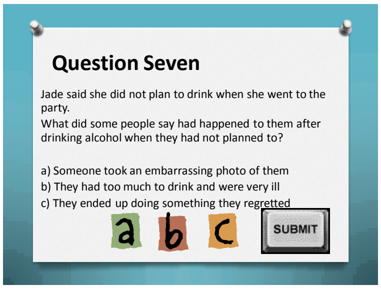
Question Seven
Jade said she did not plan to drink when she went to the party.
What did some people say had happened to them after drinking alcohol when they had not planned to?
a) Someone took an embarrassing photo of them
b) They had too much to drink and were very ill
c) They ended up doing something they regretted
a
b
c
SUBMIT

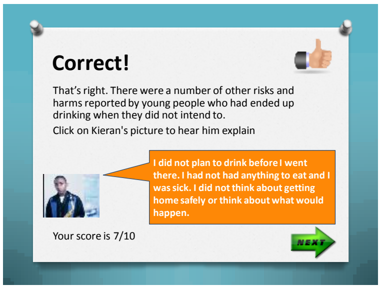
Correct!
That's right. There were a number of other risks and harms reported by young people who had ended up drinking when they did not intend to.
Click on Kieran's picture to hear him explain
I did not plan to drink before I went there. I had not had anything to eat and I was sick. I did not think about getting home safely or think about what would happen.
Your score is 7/10
NEXT

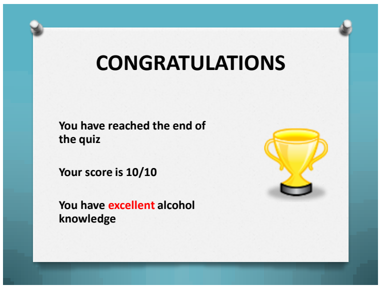
CONGRATULATIONS
You have reached the end of the quiz
Your score is 10/10
You have excellent alcohol knowledge
